# Gene Expression in Brain and Liver Produced by Three Different Regimens of Alcohol Consumption in Mice: Comparison with Immune Activation

**DOI:** 10.1371/journal.pone.0059870

**Published:** 2013-03-29

**Authors:** Elizabeth Osterndorff-Kahanek, Igor Ponomarev, Yuri A. Blednov, R. Adron Harris

**Affiliations:** Waggoner Center for Alcohol and Addiction Research, Colleges of Natural Science and Pharmacy, University of Texas at Austin, Austin, Texas, United States of America; Mayo Clinic College of Medicine, United States of America

## Abstract

Chronically available alcohol escalates drinking in mice and a single injection of the immune activator lipopolysaccharide can mimic this effect and result in a persistent increase in alcohol consumption. We hypothesized that chronic alcohol drinking and lipopolysaccharide injections will produce some similar molecular changes that play a role in regulation of alcohol intake. We investigated the molecular mechanisms of chronic alcohol consumption or lipopolysaccharide insult by gene expression profiling in prefrontal cortex and liver of C57BL/6J mice. We identified similar patterns of transcriptional changes among four groups of animals, three consuming alcohol (vs water) in different consumption tests and one injected with lipopolysaccharide (vs. vehicle). The three tests of alcohol consumption are the continuous chronic two bottle choice (Chronic), two bottle choice available every other day (Chronic Intermittent) and limited access to one bottle of ethanol (Drinking in the Dark). Gene expression changes were more numerous and marked in liver than in prefrontal cortex for the alcohol treatments and similar in the two tissues for lipopolysaccharide. Many of the changes were unique to each treatment, but there was significant overlap in prefrontal cortex for Chronic-Chronic Intermittent and for Chronic Intermittent-lipopolysaccharide and in liver all pairs showed overlap. In silico cell-type analysis indicated that lipopolysaccharide had strongest effects on brain microglia and liver Kupffer cells. Pathway analysis detected a prefrontal cortex-based dopamine-related (PPP1R1B, DRD1, DRD2, FOSB, PDNY) network that was highly over-represented in the Chronic Intermittent group, with several genes from the network being also regulated in the Chronic and lipopolysaccharide (but not Drinking in the Dark) groups. Liver showed a CYP and GST centered metabolic network shared in part by all four treatments. We demonstrate common consequences of chronic alcohol consumption and immune activation in both liver and brain and show distinct genomic consequences of different types of alcohol consumption.

## Introduction

Effects of chronic consumption of alcohol and other drugs of abuse include tolerance and dependence and these neuroadaptations arise, at least in part, from changes in gene expression [1], [2]. Several studies show changes in gene expression in autopsy brain tissue from human alcoholics [3]–[5], potentially providing therapeutic targets for new treatments for alcoholism. However, drug development requires testing in animal models and there is only limited information on brain gene expression changes in rodent models of chronic alcohol consumption [1]. For the Preferring (P) line of rats, three studies of ethanol consumption found changes in gene expression that often varied between brain regions [6]–[8]. Mice are widely used to study alcohol consumption but analyses of brain gene expression profiles following chronic alcohol drinking are remarkably limited for mouse models [1], [9], [10] and there are no direct comparisons of genomic changes in different animal models. One theme that has emerged from studies of gene expression in human alcoholism is the role of neuroimmune genes [3], [11], [12] and differential expression of this category of genes is also seen in mice with genetic predisposition for high alcohol consumption [13]. In addition, activation of the innate immune system with LPS and the availability of chronic ethanol both increase alcohol consumption [14], [15]. This raises the question of which, if any, of the rodent models of excessive alcohol consumption show changes in gene expression, neuroimmune genes, in particular, that might be similar to human alcoholism and similar to the immune activation produced by LPS. A major consequence of chronic alcohol consumption is altered liver function, often accompanied by steatosis and other alcoholic liver injuries. A few studies have examined changes in liver gene expression produced by administration of alcohol by chronic intragastric infusion or consumption of a liquid diet [16], [17], but there is a paucity of studies of liver gene expression profiles in any of the mouse models of voluntary alcohol consumption.

The present study was designed to provide a direct comparison of the effects of chronic alcohol consumption from three different mouse models on brain (prefrontal cortex, PFC) and liver gene expression as well as to identify the ethanol treatment whose effects were most similar to the immune response produced by LPS. PFC was chosen because it is commonly used in studies of gene expression in human alcoholics [3] and is a brain region important for consequences of chronic alcohol consumption [18], [19]. We chose the continuous two bottle choice (Chronic) test because it is probably the most widely used model of mouse alcohol consumption [20], [21], the every other day or chronic intermittent (CI) model because it promotes high intake and is gaining popularity for medication development [22], [23] and the limited access drinking in the dark (DID) test because it produces high blood ethanol levels and is a model of binge drinking [15]. Inflammatory processes and activation of innate immunity is established as a critical component of alcoholic liver disease [24], [25] and is also emerging as an important determinant of alcohol effects on brain and on alcohol consumption [14], [26], [27]. Thus, it was of interest to compare immune activation following injection of lipopolysaccharide (LPS), which increases alcohol consumption, to the alcohol treatments. Microarrays were used to profile gene expression in two tissues (PFC and liver) from each of the four groups (three alcohol treatments, one LPS treatment), followed by data analysis to determine changes in gene expression produced by each treatment, the overlap of changes between treatments, pathway analysis of the gene networks and cellular enrichment of the differentially expressed genes. These data show distinct changes in gene expression in PFC and liver as well as among the treatments, but also show overlap between several of the treatments (notably, CI and LPS) and provide signaling networks that may mediate some of the consequences of chronic alcohol exposure. Comparison of gene expression changes among these four treatments and with published data on other animal models and human alcoholics provides convergent validity for the role of several signaling pathways in excessive alcohol consumption.

## Materials and Methods

### Ethics Statement

All procedures were approved by the University of Texas at Austin Institutional Animal Care and Use Committee (mouse protocol number AUP-2010–00028) and adhered to NIH Guidelines. The University of Texas at Austin animal facility is accredited by the Association for Assessment and Accreditation of Laboratory Animal Care.

### Animals for Ethanol Studies

Studies were conducted in adult drug-naïve C57BL/6J (B6) female mice from a colony maintained at the University of Texas. C57BL/6J (B6) female mice were utilized, as they are known to voluntarily consume more ethanol than males[28]–[30]. Original breeding pairs were purchased from Jackson Laboratories (Bar Harbor, ME) and mated at 8 weeks of age. Mice were initially group-housed in standard polycarbonate shoebox cages (four-five per cage), then moved to individual cages and allowed to acclimate for 1 week prior to treatment. DID animals were given an additional two-week acclimation period to adjust to the reverse light cycle (see below). Food (Prolab RMH 1800 5LL2 chow, TestDiet, Richmond, IN) and water were provided *ad libitum*, except as noted below. The colony rooms and testing rooms were maintained at an ambient temperature of 21±1°C, humidity (40–60%), and centrally controlled ventilation (12–15 cycles/h with 100% exhaust). All treatment and control groups consisted of 10 animals, except the treated CI group, which contained 11 animals. A 20% (v/v) ethanol solution was used for all studies. Ethanol and water bottles were weighed daily and animals were weighed every four days.

### Chronic Ethanol

The Chronic ethanol treatment group included 10 each treated and control mice maintained on a 12∶12 light/dark light cycle (lights on at 07∶00 AM). Mice were approximately 3 months old at the beginning of experiments. Water and ethanol were continuously available for a 30-day period and bottle positions were changed daily to control for position preference. Control animals received only water.

### Chronic Intermittent

The CI treatment utilized 11 treated and 10 control animals and employed similar conditions to those used for the Chronic study, except that ethanol was only available every other day. On days that ethanol was available, bottle positions were alternated to control for position preference. Mice were approximately 3 months old at the beginning of experiments. The total duration of the CI experiment was determined by the total amount of ethanol consumed, which was matched with the total amount of ethanol consumed in the Chronic paradigm. In the Chronic drinking paradigm, the average total ethanol intake for all mice was 406 g/kg. Thus, the CI study was concluded when the average total ethanol intake for all mice reached a similar level: 409 g/kg after 29 days of drinking. Since these animals only had access to ethanol every other day, the total length of the study, including drinking and non-drinking days, was 57 days. CI animals were maintained on a 12∶12 light/dark light cycle (lights on at 07∶00 AM). Control animals received only water. Data from all animals was used for ethanol consumption analyses. For array analysis, one treated animal was omitted (chosen at random) from microarray experiments in order to maintain continuity of sample sizes across treatment paradigms.

### Drinking in the Dark

The DID protocol was also employed as it achieves pharmacologically significant levels of ethanol [31]. For this treatment, animals (approximately 2 months old at the beginning of experiments) were maintained on a 12∶12 reverse light/dark cycle (lights on at 07∶00 PM.) Starting 3 hr after lights off, water bottles were replaced with bottles containing a 20% ethanol solution. The ethanol bottles remained in place for either 2 (first 3 days) or 4 hr (day 4) and then were replaced with water bottles. Except for this short period of time of ethanol drinking, mice had unlimited access to water. This procedure was repeated for 36 consecutive days. Control animals received only water.

### Lipopolysaccharide Treatment

Lipopolysaccharide (LPS) studies were also conducted in adult (2 months of age at the beginning of experiments) drug-naïve C57BL/6J (B6) female mice housed under the same conditions as those used in the ethanol studies. LPS is an endotoxin known to produce a strong immune response in mice that is characterized by a number of symptoms including decreased water consumption and weight loss which return to control levels after a few days [14], [32]. Moreover, ethanol consumption is increased in both male and female mice after 1 or 2 LPS injections [14]. LPS (strain O111:B4, Sigma Chemical Co., St. Louis, MO) dissolved in saline was injected at a dose of 1 mg/kg i.p. in volume 0.1 ml/10 g of body weight. A second LPS injection was made one week after the first injection and mice were euthanized one week after the second injection. This is based on our previous studies using two injections of LPS separated by one week with drinking tests beginning one week after the last injection [14]. Treatment and control groups contained 10 animals each. Control animals received saline injections in lieu of LPS.

### Tissue Harvest and RNA Isolation

Tissues were harvested between noon and 2 PM, 20–24 hours after the last exposure to ethanol or 1 week after the second LPS injection. (DID animals, which were housed on a reverse light cycle, were euthanized during the dark period whereas all other animals were euthanized during the light cycle.) To expedite the procedure and minimize RNA degradation, different individuals performed brain and liver dissections concomitantly. Animals were euthanized by cervical dislocation and brains were removed and placed in a petri dish on ice. After removal of olfactory bulbs, PFC was dissected by cutting the foremost 2 mm of the cortex on each side, at an approximate 50-degree angle from the midline of the brain. Liver samples, approximately 100 mg each, were removed from the lower lobes of the liver. All samples were immediately frozen in liquid nitrogen and stored at −80°C until use.

Total RNA was isolated according to manufacturer’s instructions using the mirVana**™** miRNA Isolation kit (Ambion, Austin, TX). Total RNAs were DNase treated (Turbo DNA-free**™**, Ambion, Austin, TX), quantified on a NanoDrop 1000 spectrophotometer (Thermo Fisher Scientific Inc., Rockford, IL), assessed for quality on an Agilent 2100 Bioanalyzer (Agilent Technologies, Santa Clara, CA) and amplified/biotin-labeled using the Illumina® TotalPrep**™** RNA Amplification kit (Ambion, Austin, TX). Aliquots of labeled cRNA were sent to the Yale Center for Genome Analysis (West Haven, CT) where they were hybridized to Illumina® MouseRef-8 v2 Expression BeadChips (Illumina, Inc., San Diego, CA) according to manufacturer protocols. As each beadchip contains 8 independent arrays, samples were hybridized to beadchips in a group counter-balanced format to minimize batch effects. Each array was hybridized with material obtained from a single animal. Each expression array contains approximately 25,600 transcripts representing over 19,100 unique genes. Transcript abundance was measured by fluorescent intensity after scanning. Microarray data have been submitted to the NCBI Gene Expression Omnibus (GEO) (http://www.ncbi.nlm.nih.gov/geo/) under accession no GSE42789.

Although historically it has been standard practice to verify a subset of array-generated gene expression changes using qRT-PCR, we did not include such confirmation in the present study. We have used Illumina platforms (including the one used in this study) extensively and “validated” them with independent qRT-PCR experiments in the past. The level of correspondence between the microarray and RT-PCR results exceeds 80% [11], [33], [34].

### Statistics and Informatics

Ethanol consumption data are presented as mean ± S.E.M. Microarray data were analyzed using the R statistical environment [35] and Microsoft Excel (2011). Only genes with a detection p value ≤0.05 and present on >80% of arrays were utilized in the analysis of each tissue dataset. Variance stabilization transformation [36] and quantile normalization [37] were used to pre-process the data in Lumi [38]. Expression value outliers were removed using Grubbs’ test with a critical value of 2.21 or 2.29, depending on the number of analyzed arrays. One sample (a Chronic PFC control) clustered separately in the Lumi outlier detection tree and had more than 5% outlier genes and was thus removed from the analysis. Limma [39] was used to fit a linear model for each gene and detect differentially expressed genes using an empirical Bayes method. Fold changes in gene expression are given as change in treated relative to control. Significant overlap of differentially expressed genes among pairs of studies was assessed with a Bonferroni-corrected Chi-square goodness of fit test. The Pearson product-moment correlation was used to evaluate correlation of ethanol consumption with individual gene expression values.

Each study was assessed for the presence of either brain or liver cell type-specific genes, depending on the tissue being evaluated. Cell type-specific datasets were downloaded from the Journal of Neuroscience website: [40] (neuron, astrocyte, oligodendrocyte), the Nature Neuroscience website: [41] (microglia) or the World Journal of Gastroenterology website: [42] (hepatocyte, hepatic stem cell and Kupffer cell). The criterion for brain cell type specificity was a four-fold enrichment [11]. A ten-fold enrichment was the primary criterion used for liver cell type specificity. (See [42] for detailed information.) Gene symbols from our data and the cell-type specific data sets were converted to the currently accepted gene symbol using the Database for Annotation, Visualization and Integrated Discovery (DAVID v6.7 [43], [44]). Converted gene symbols were compared to identify cell-type specific genes present in our data sets. Mean t values were calculated for the cell-type specific genes identified in each study/tissue and a z test was used to determine whether each of these values was equivalent to zero. A Bonferroni correction was applied to p values to correct for multiple comparisons within a tissue.

Ingenuity Pathways Analysis (Ingenuity® Systems, www.ingenuity.com) was used to assess microarray data for overrepresentation of known gene networks and biological functions. All detected genes in a data set were used as the reference set; cutoffs for fold change and the associated p value were 1.2 and 0.05, respectively. Default settings were used for all other parameters. For network analysis, network eligible molecules (data set molecules that interact with the Ingenuity Knowledge Base) were used as “seeds” to generate networks whose molecules’ interconnectedness is maximized relative to their connectedness with all molecules in the knowledge base. Network scores were calculated by taking the negative log_10_ of the p value computed with Fisher’s exact test. Functional analysis identified the biological functions and/or diseases that were most significant to each data set. A right-tailed Fisher’s exact test was used to calculate the p-value determining the probability that each biological function and/or disease assigned to that data set is due to chance alone.

## Results

### Ethanol Exposure

**Figure 1:**
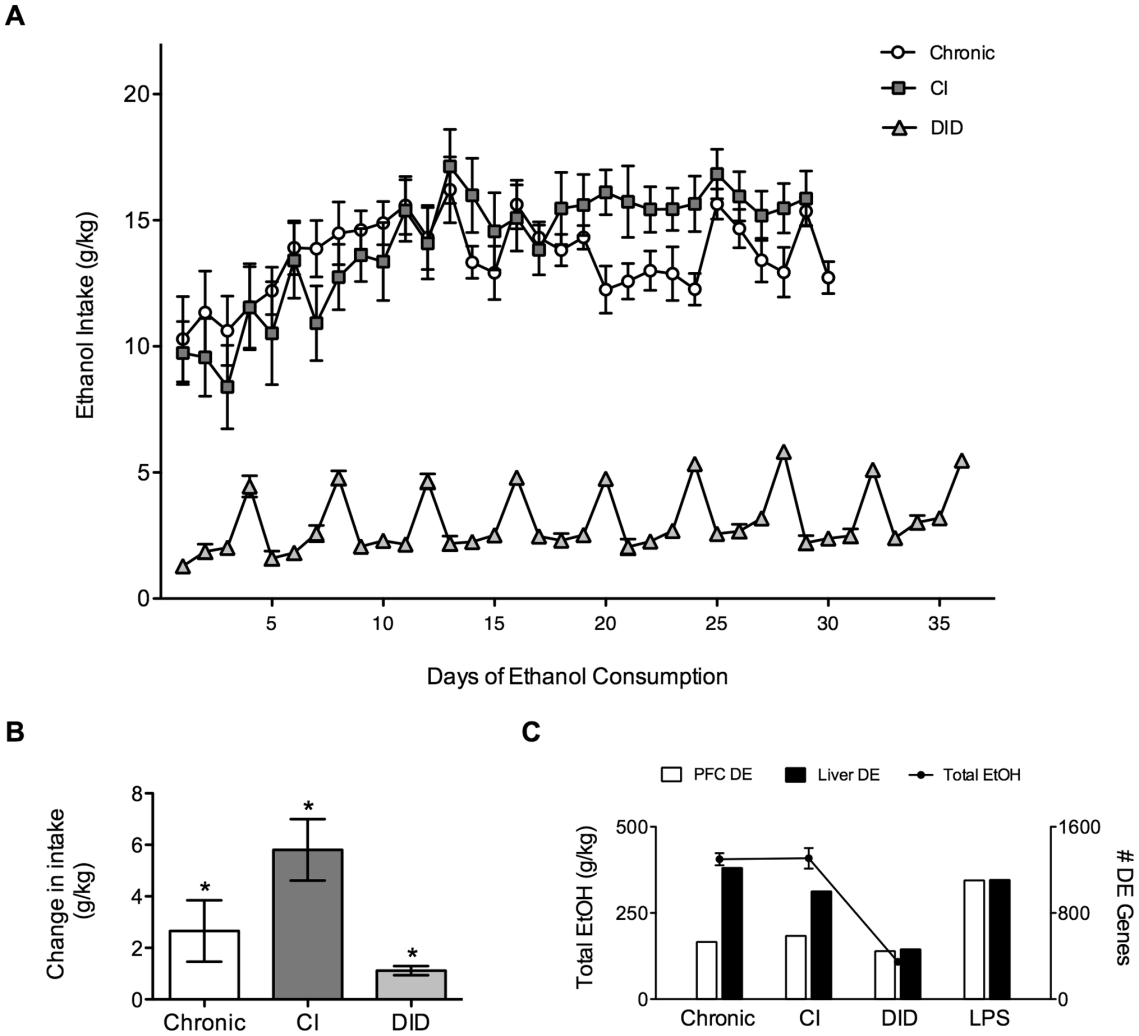
Ethanol consumption and gene expression changes in liver and prefrontal cortex. (A) Average daily ethanol intake for three ethanol treatments. The Chronic treatment (open circles) is continuous two bottle choice drinking, CI is chronic intermittent two bottle choice with access to alcohol every other day and DID is a limited daily access (2 hr or 4 hr) to alcohol. Only data for days of ethanol consumption are shown. (B) Change in ethanol intake between the first and last 4 days of each treatment (mean ± SEM). Asterisks identify a significant change in ethanol intake (paired t test, p≤0.05). (C) Total ethanol consumed (average of all animals) and the number of genes differentially expressed (DE, p<0.05) in PFC and liver in each treatment (open bars are prefrontal cortex, filled bars are liver). Values are mean ± SEM, for n = 10 (Chronic and DID), n = 11 (CI). For some values, error bars are smaller than the symbols.

Daily ethanol consumption by mice in both the Chronic and CI treatment groups increased steadily during the first two weeks, then remained fairly stable for the remainder of the study (Figure 1A). During the last 10 days of ethanol exposure, mice in the CI group consumed more ethanol than those in the Chronic group (CI, n = 11, mean = 16 g/kg/day; Chronic, n = 10, mean = 13 g/kg/day; student’s t test, p = 0.047). Mice in the DID paradigm consumed greater quantities of ethanol during the 4 hour exposures than the 2 hour exposures but did not reach the total consumption levels attained in the other ethanol treatment paradigms. These results are consistent with previous publications [31], [45]. In all three models, mice increased their consumption of ethanol over time as indicated by a significant difference between the first and last four sessions (Figure 1B). (Percent increase and paired t test, Chronic, 24% increase, p = 0.05; CI, 59% increase, p = 6.4E–04; DID, 46% increase, p = 1.4E–04).

### Gene Expression Changes

For all groups, more genes were expressed in PFC than in liver, but liver showed both a larger number and percentage of significantly regulated (Differentially Expressed, DE) transcripts (Table 1). Total ethanol consumption paralleled gene regulation in that treatments (Chronic and CI) evoking greater ethanol consumption also yielded a larger number of regulated transcripts than the lower consumption test (DID), especially in the liver (Figure 1C). This likely reflects the increased metabolic load imposed on the liver with increasing amounts of ethanol. The LPS injections strongly perturbed the PFC transcriptome and LPS-treated animals exhibited almost twice as many DE genes as the Chronic and CI treatments (Table 1). In liver, the LPS and ethanol treatments generated comparable numbers of DE genes.

**Table 1:** Total number of transcripts detected and differentially expressed in liver and PFC.

Study	Tissue	Total # Detected Transcripts	# DE* transcripts (p<0.05)	% DE* transcripts (p<0.05)
Chronic	PFC	12125	531	4.4%
Chronic	Liver	10068	1219	12.1%
CI	PFC	11269	587	5.2%
CI	Liver	9561	1001	10.5%
DID	PFC	11473	445	3.9%
DID	Liver	8924	463	5.2%
LPS	PFC	11508	1103	9.6%
LPS	Liver	9550	1107	11.6%

* DE = differentially expressed.

**Figure 2:**
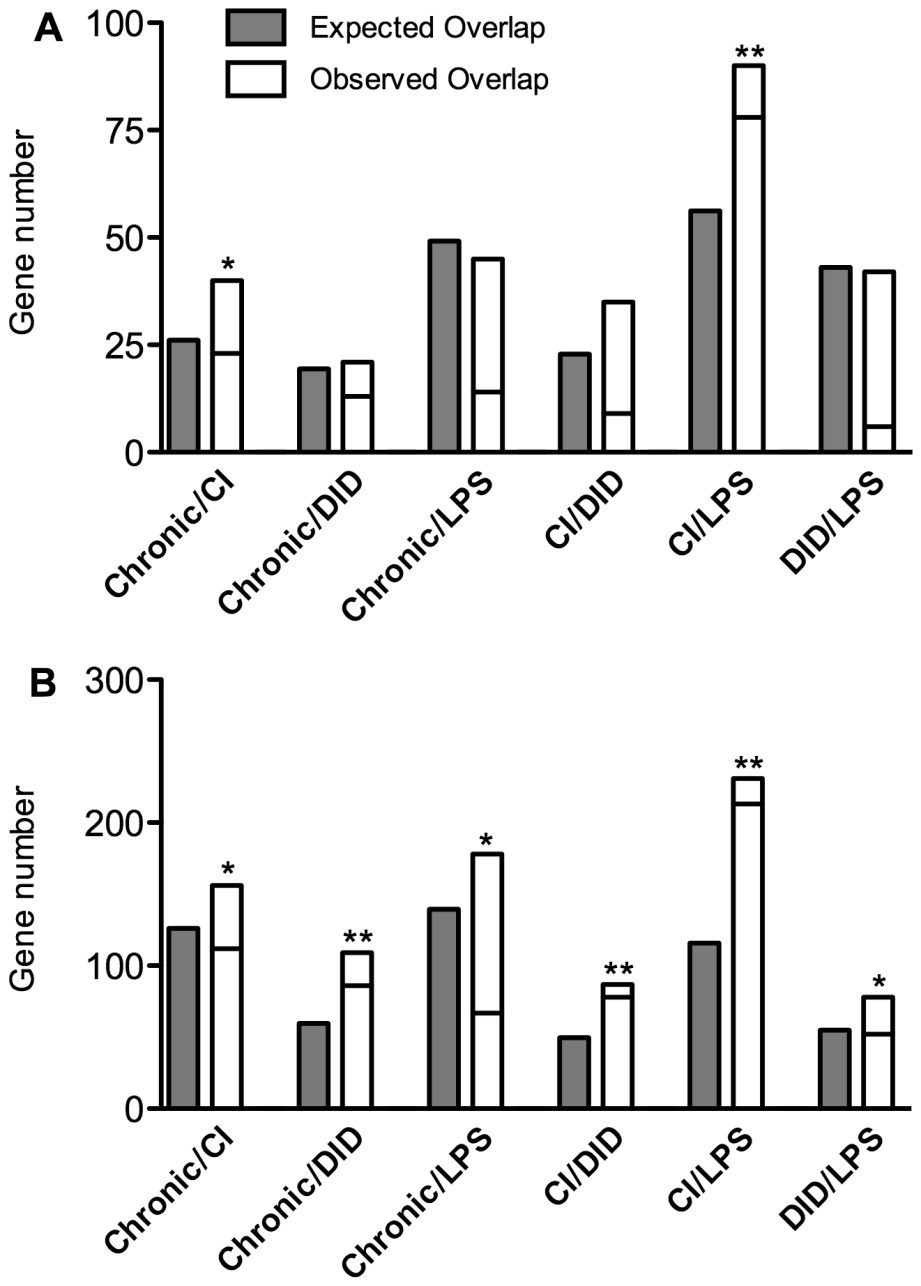
Overlap of differentially expressed genes in pairs of studies for each tissue. (Panel A, PFC; Panel B, liver). Filled bars show the number of genes expected to be differentially expressed in both compared studies. Open bars show the observed number of shared, differentially expressed genes, with a horizontal line indicating the observed number of genes regulated in the same direction. Observed values are significantly greater than expected by chance (Bonferroni-corrected Chi square goodness-of-fit) at p<0.05 (*) and p<0.0001 (**).

Overlap of regulated transcripts in pairs of studies for each tissue type was evaluated by comparing probe IDs of DE genes from each pair of studies (Figure 2). In PFC, there was significant overlap between Chronic and CI and between CI and LPS, but no overlap for the other pairs of treatments. In liver, all pairwise comparisons showed an overlap of DE genes that was significantly greater than expected by chance for all study comparisons (Figure 2). Moreover, the majority of shared DE genes tend to be regulated in the same direction in both studies (Figure 2). LPS and CI treatments displayed the most similarly regulated transcripts in PFC (78 transcripts) and in liver (213 transcripts).

### Correlation of Gene Expression and Alcohol Consumption

Using data from individual mice, total ethanol consumption was correlated with PFC gene expression (p<0.05) for 753 and 702 transcripts in Chronic and CI treated animals, respectively (Table S1). Likewise, liver from Chronic animals contained a large number (942) of transcripts whose expression levels were significantly correlated with total ethanol consumption (Table S2). Other treatments gave smaller numbers of correlated transcripts, and about 400 transcripts were significantly correlated with total ethanol consumption in either liver or PFC from DID-treated animals and liver from CI treated animals (Tables S1 and S2).

### Cell-type Analysis of Expression

**Figure 3:**
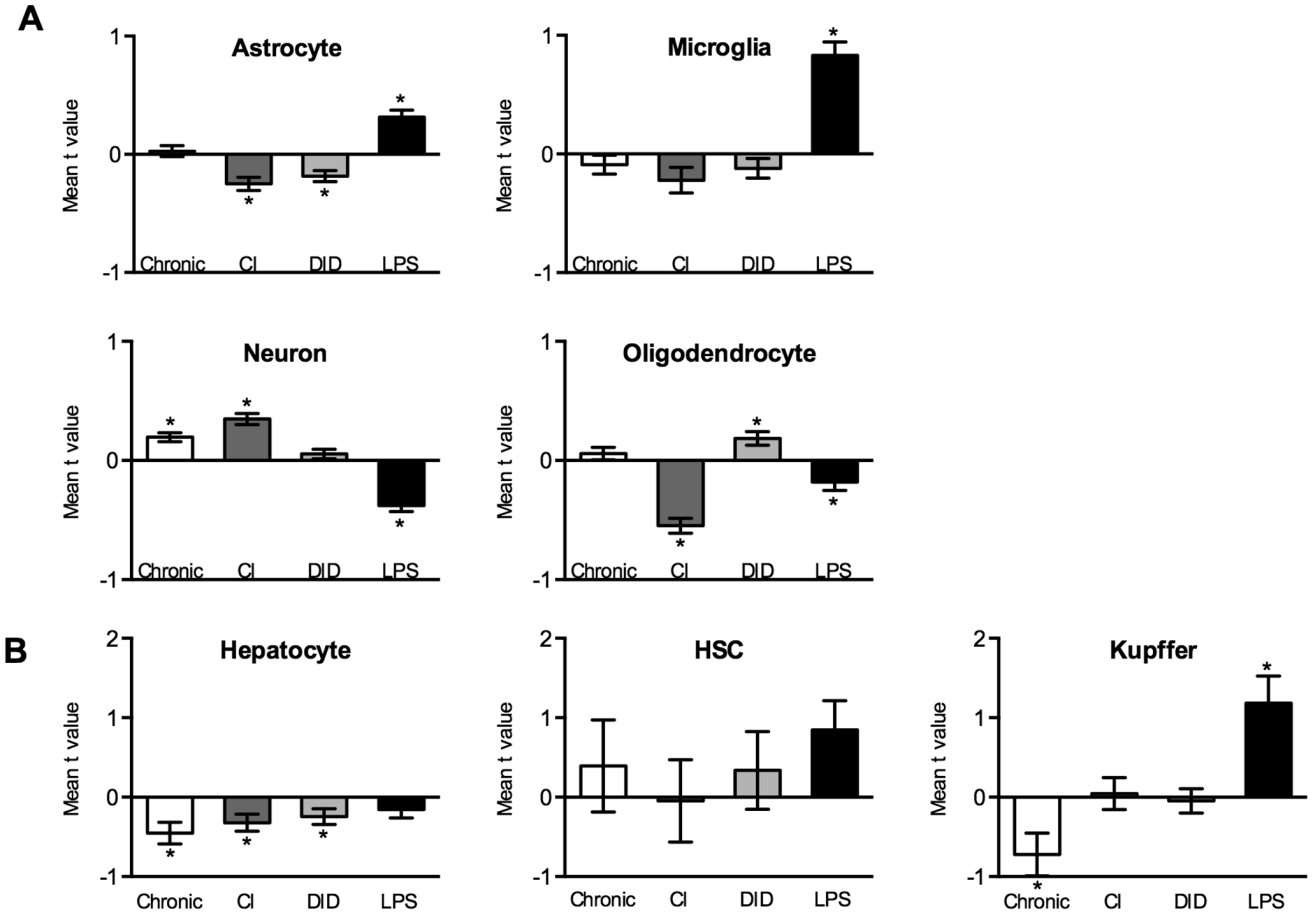
Mean t values of cell-type specific genes expressed in PFC and liver. Bars show mean t values (+/− SEM) of cell-type specific genes identified in each treatment. Asterisks identify cell-type specific t-means that are significantly different (by z test, Bonferroni-corrected, p<0.05) from the mean t value of all cell-type specific genes [n] detected in the given study. A. PFC (Astrocyte, n = 451–468; Microglia, n = 144–156; Neuron, n = 695–750; Oligodendrocyte, n = 264–297) B. Liver (Hepatocyte, n = 210–226; HSC, n = 11–15; Kupffer, n = 21–32).

We next used cell-type specific databases to ask which treatments might disproportionately affect genes enriched in different types of cells. The clearest finding is that LPS treatment increased expression of genes enriched in PFC microglia and liver Kupffer cells (Figure 3). The alcohol treatments produced modest changes in cell-type specific expression in PFC (Figure 3A). It should be noted that these analyses are based on all genes with cell-type enrichment whereas the data in Figure 2 are derived from all differentially expressed genes, thus the overlaps seen in Figure 2 (e.g., CI and LPS) are not prominent in the cell-type analysis.

### Ingenuity Pathway Analysis (IPA)

**Figure 4:**
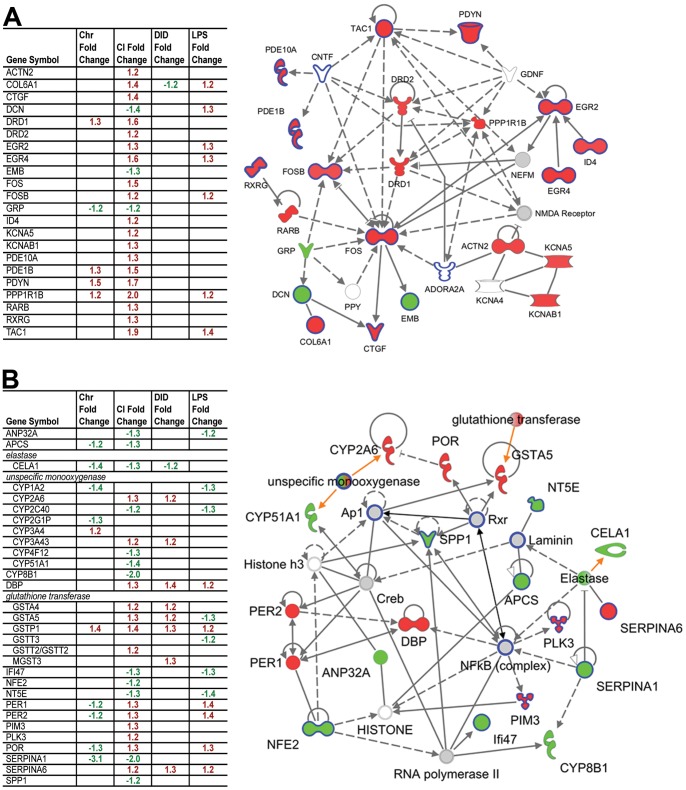
Gene networks derived from Chronic Intermittent (CI) data. Red and green fill indicate up- and down regulation, respectively in treated animals relative to controls (fold change ≥1.2, p<0.05). Gray fill indicates gene was not differentially expressed using these thresholds. White fill indicates genes not detected in our data, but added to the network due to their connectedness with other genes. Orange arrows point to members of a gene family. Solid lines indicate direct relationships; hashed lines are indirect relationships. Dark blue outlines identify genes that regulate cytokines or are regulated by cytokines or LPS. Tables show the fold changes of network genes up regulated (red) and down regulated (green) in all four data sets at p<0.05. Shapes represent molecule types. Genes are identified with human gene symbols. See Figure S1 for legend of molecule shapes. A. Neuronal network derived from PFC. B. Top network derived from liver.

IPA identified the CI treatment as containing the most statistically significant networks and biological functions in PFC (Table S3). Neurological disease and genetic disorders were the top identified biological functions and were also represented among the functions of the top five networks, thus we chose to investigate these annotation groups further. Since IPA functional groups do not represent a known biological network *per se*, we used the top biological function results to derive our own network. Briefly, the two genetic disorder-related annotation groups containing the largest numbers of molecules (“genetic disorder” and “Huntington’s disease”) were combined and their molecules connected by knowledge base relationships to form a preliminary network. This network was “grown” once, adding a limited number of related molecules with high specific connectivity to the network. The resulting PFC network contains a variety of genes involved with dopaminergic signaling, as well as genes related to immune function (Figure 4A). Notably, when data from each of the other studies are overlaid on this CI dopaminergic network, similarities between LPS and CI treatments are revealed. These similarly regulated genes are identified in the tables to the left in Figure 3.

The top network identified in CI liver (Figure 4B) was associated with drug metabolism, glutathione depletion and behavior (Table S3). In contrast to the PFC network detailed above, this network showed many similarities among liver from all four treatments, suggesting a more generic response in liver than PFC.

## Discussion

One of the most surprising findings from this work is the distinct effects of the three alcohol treatments on the PFC transcriptome. Despite consuming the same total amount of alcohol, the Chronic and CI groups showed many differences in gene expression. The CI test was originally derived from studies in rats that showed a marked escalation of alcohol consumption with every other day access [23] and CI, but not Chronic, drinking is inhibited by chlorzoxazone [22] indicating differences in the neurobiological mechanisms underlying these two drinking tests. In mice, we found the largest increase in alcohol consumption for the CI group and smaller increases for Chronic and DID drinking during the exposure period (approximately 30 days), which is generally consistent with recent publications [15], [45]. Perhaps because of the greater escalation of consumption in the CI group, the neuroadaptive consequences of every other day access appear to be distinct from continuous access. For example, our cell type analysis indicated changes in expression of genes enriched in oligodendrocytes by CI, but not by Chronic. Most rodent models of excessive or escalating alcohol consumption incorporate intermittent access, as reviewed by Becker (2013). The mechanisms are not well defined, but may involve physical dependence and a withdrawal syndrome during deprivation as suggested by studies showing increased alcohol withdrawal and consumption after repeated alcohol exposure and withdrawal [46]–[48]. We also included the DID test as a model of binge drinking which produces higher blood ethanol levels than Chronic or CI, but for only a short period of time each day [15]. This resulted in the fewest changes in gene expression of all the treatments, particularly in liver where the number of gene changes produced by DID were less than half the number produced by the other treatments, likely reflecting the much lower total intake of ethanol in the limited access DID test.

Another notable result from this study is that the LPS treatment produced twice as many changes in gene expression in the PFC as the CI or Chronic alcohol treatments and a similar number of changes in liver as alcohol consumption, even though LPS was given a week before the end of the experiment. This LPS treatment produces a persistent increase in alcohol consumption and a decrease in the firing of midbrain dopamine neurons [14], which may be consequences of some of the observed changes in gene expression. Cell type analysis showed that LPS altered expression of genes enriched in two macrophage derived cell types: microglia and Kupffer. Also remarkable is the overlap of differentially expressed genes from alcohol treatments and LPS. For both PFC and liver, the highest degree of overlap between treatment groups was for CI and LPS. For the CI gene networks assembled for both PFC and liver, the overlap of genes was greater for LPS than for the ethanol treatments (Chronic or DID). This may reflect emerging evidence that ethanol promotes proinflammatory signaling in brain and liver by increasing TLR4 activation and cytokine cascades [12] ^,^ [24], [25], [49]–[51]. LPS acts on TLR4 and thus has the potential to share activation of TLR4 function with ethanol, especially in the liver. It is controversial as to whether LPS can cross the blood brain barrier and whether its effects on brain function are likely to be secondary to release of cytokines from liver and other peripheral tissues or a direct effect on brain microglia and endothelia [52], [53]. It is interesting to note that chronic alcohol abuse can compromise the gut and allow leakage of LPS into the systemic circulation and this is important for development of alcoholic liver disease [24]. Although we did not combine LPS and alcohol exposures, our studies showing overlap between the transcriptome modifications produced by the separate treatments supports the proposal that LPS can enhance some of the biological actions of ethanol, and vice versa [49], [50].

A novel aspect of this work is that our pathway analysis of the PFC transcriptome highlights a complex of genes related to dopaminergic neuroplasticity that have been implicated in addiction to ethanol and other drugs. This network has hub genes including PPP1R1B (DARPP-32), DRD2, DRD1, FOS and FOSB (Figure 4A), all of which are supported as key components of the progressive and persistent changes in synaptic plasticity that arise from chronic drug exposure [54]. In particular, DARPP-32 regulates the alcohol sensitivity of NMDA receptors [55] and contributes to genetic differences in alcohol consumption in the AA/ANA rats [56] as well as having a role in actions of many drugs of abuse [57]. This network also includes PDYN (prodynorphin) which is of interest as it regulates alcohol consumption through dopamine neurotransmission [58] and PDYN polymorphisms are linked to alcoholism [59], [60] as well as heroin and cocaine addiction in humans [61]. Genetic manipulation in PDYN or κ-opioid receptors also supports the importance of this network in regulation of alcohol consumption in mice [62], [63]. Two phosphodiesterases, PDE10A and PDE1B, are part of the network and PDE10A regulates DARP-32 phosphorylation [64]. PDE10A was one of the top PFC genes in a study [10] of individual differences in alcohol drinking in mice and expression of PDE10A is increased by stress, which appears to be important for stress-induced increases in alcohol consumption in rats [65]. Our PFC network is based on changes in expression of 22 genes produced by CI drinking, and five of the genes (including DRD1, PDYN and PPP1R1B) are also changed by Chronic consumption and seven are changed by LPS treatment. For all differentially expressed genes, there was significant overlap between CI and Chronic as well as CI and LPS. CI and LPS share the behavioral feature of increasing alcohol consumption. In view of the representation of some components of the dopamine network in the LPS group, it is important to note that a similar LPS treatment produces a decrease in the firing of brain dopamine neurons [14].

For liver, a network was constructed that features cytochrome monooxygenases, glutathione transferases and circadian genes (PER1, 2) together with a number of transcription factors (Creb, NFkB, DBP, AP1, NFE2) as hub genes. This network was based on the CI treatment but many of the genes are also changed by Chronic, DID and LPS treatments. For all differentially expressed genes, there was significant overlap among all alcohol treatments and for LPS with all alcohol groups. This demonstrates that although some of the changes in the liver transcriptome may be due to the metabolism of large amounts of ethanol in this tissue, many of the effects are mimicked by LPS, which suggests that a component of alcohol action on liver is due to immune activation. This is consistent with the key role of inflammation in alcoholic liver disease [24], [25].

Several groups have published changes in brain gene expression related to alcohol and we have compared their data with the present study in Table S4. The studies included are a comparison of differences in genetic predisposition to alcohol consumption in mice [13], individual differences in alcohol consumption in mice [10], consumption of alcohol in alcohol-preferring rats [6], human alcoholics [3], [11], gene expression changes in three mouse models of ethanol consumption [66] and gene expression changes in mice after chronic intermittent ethanol exposure [67]. Table S4 also contains a comparison of our data with LPS-induced gene expression changes in mouse astrocytes [68]. Several genes from our dopamine-centric pathway (Figure 4A) are found in multiple studies. For example, DRD2, EGR2, KCNA5 and PDE10A were all found in by Mulligan et al. [13] and, as noted above, PDE10A was one of the top genes in the Wolstenholme et al [10] study. FOS, a main hub gene in our pathway analysis, was also changed in the Bell et al. [6] data and two other genes in our pathway, PDYN and DRD1, were changed in the Contet et al. [66] study. B2M was changed by LPS treatment in the present study, was one of the top genes in Mulligan et al. [13] and was also changed in the Melendez et al. [67] study of chronic intermittent ethanol exposure in mice. This gene is related to inflammatory processes and is changed in astrocytes in response to LPS exposure [68]. Notably, deletion of B2M in mice reduces alcohol consumption [26]. Of the 137 genes from the present study listed in Table S4, 48 were differentially expressed in the Ponomarev et al. [11] study of prefrontal cortex of human alcoholics and 25 were changed in the Liu et al. [3] data.

In summary, comparison of three different models of voluntary alcohol consumption in mice shows changes in gene expression in PFC that reveal some similarities, as well as many differences, among the models. Effects on gene expression in liver are more similar for the treatments. Gene expression was also studied one week after injection of LPS and this treatment showed many changes that were similar to ethanol consumption, particularly the CI model. Our data suggest DID is the weakest model of chronic consumption/dependence based on the number of changes in gene expression in PFC, but it remains a relevant model of binge/initiation of drinking which involves activation of brain structures other than PFC. The CI paradigm represents a good model of the neuroimmune component of chronic alcohol consumption as it is most similar to LPS and it reflects some aspects of the strong immune component found in gene expression studies of PFC in human alcoholism. We also provide the first evidence for a connection between immune response, ethanol intake and dopamine signaling in PFC and the CI paradigm provides an approach for future studies of these interactions. It is important to note that both LPS treatment and the CI model promote an escalation of drinking, supporting the emerging concept that ethanol consumption is regulated by neuroimmune signaling [14], [26], [27].

## Supporting Information

Figure S1 **Legend of molecule shapes used in gene network diagrams.**
(TIF)

Table S1 **PFC differential expression and ethanol correlation data.**
(XLSX)

Table S2 **Liver differential expression and ethanol correlation data.**
(XLSX)

Table S3 **Most significant network functions, biological functions and canonical pathways detected by Ingenuity pathway analysis.**
(XLSX)

Table S4 **Top genes differentially expressed in the present study and at least one other ethanol- or LPS-related study.**
(XLSX)
