# Supplementary figures and images for: Gene Expression in Brain and Liver Produced by Three Different Regimens of Alcohol Consumption in Mice: Comparison with Immune Activation
Source: PLoS One. 2013 Mar 29;8(3):e59870. doi: 10.1371/journal.pone.0059870 (PMC3612084; doi:10.1371/journal.pone.0059870)

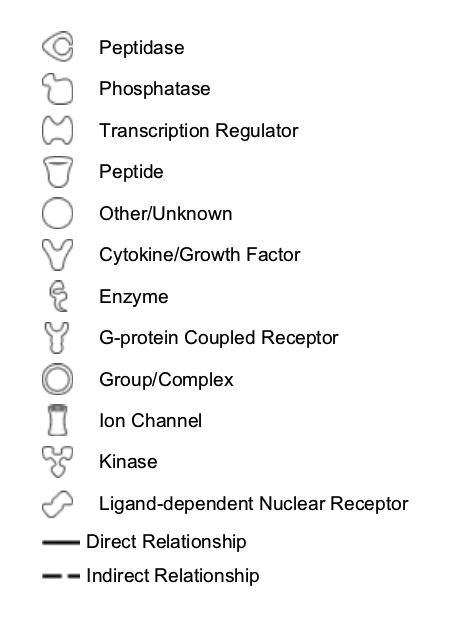

Supplement: Figure S1 — Legend of molecule shapes used in gene network diagrams. (TIF) [file pone.0059870.s001.tif]
